# *Potamophylaxkosovaensis* sp. nov. (Trichoptera, Limnephilidae), a new species of the *Potamophylaxwinneguthi* species cluster from the Ibër River Basin in Kosovo

**DOI:** 10.3897/BDJ.12.e121454

**Published:** 2024-04-05

**Authors:** Halil Ibrahimi, Astrit Bilalli, Donard Geci, Linda Grapci Kotori

**Affiliations:** 1 Department of Biology, Faculty of Mathematics and Natural Sciences, University of Prishtina “Hasan Prishtina”, Prishtina, Kosovo Department of Biology, Faculty of Mathematics and Natural Sciences, University of Prishtina “Hasan Prishtina” Prishtina Kosovo; 2 Faculty of Agribusiness, University “Haxhi Zeka”, Peja, Kosovo Faculty of Agribusiness, University “Haxhi Zeka” Peja Kosovo

**Keywords:** freshwater biodiversity, aquatic insects, Western Balkans, Kopaonik Mountains, microscale endemic species, taxonomy

## Abstract

**Background:**

The *Potamophylaxwinneguthi* species cluster comprises species with limited distribution, currently documented from Kosovo, Serbia, Bosnia and Herzegovina and Bulgaria.

**New information:**

In this paper, we describe a new species, *Potamophylaxkosovaensis* sp. nov., discovered in two tributaries of the Ibër River Basin, within the Kopaonik Mountains of the Republic of Kosovo. Morphologically, males of this species closely resemble those of *P.idliri* Ibrahimi, Bilalli & Kučinić, 2022 from Serbia's Jastrebac Mountain and *P.humoinsapiens* Ibrahimi & Bilalli, 2023 from the Sharr Mountains in the Republic of Kosovo. However, the new species can be distinguished by its smaller aedeagus, thicker and differently-shaped parameres, as well as distinctive patterns and elongated spines on the parameres. Notably, this species is restricted to spring areas, indicating sensitivity to water pollution and habitat degradation. Additionally, we provide a list of caddisfly species found in sympatry with the new species.

*Potamophylaxkosovaensis* sp. nov. is the third known species within the *Potamophylaxwinneguthi* species cluster, identified in the Republic of Kosovo.

## Introduction

The usage of aquatic insects in the Republic of Kosovo, but Western Balkans as well, for the assessment of quality and ecological integrity of freshwaters is still low (e.g. Ibrahimi et al. 2021a, Bilalli et al. 2022, Pešić et al. 2022. This stems mostly from the lack of knowledge of taxonomy and ecology of freshwater insects in this area. In this context, the intensified investigations of caddisflies in the Balkan Peninsula throughout recent years have improved significantly the taxonomic knowledge of this aquatic insect order and has also led to the discovery of numerous new species, particularly in the mountainous regions, unveiling several microscale endemic species (Ibrahimi et al. 2015, Ibrahimi et al. 2016, Oláh et al. 2017, Hinić et al. 2020, Ibrahimi et al. 2021b, Oláh et al. 2022, Ibrahimi et al. 2023, Vitecek et al. 2015, Valladolid et al. 2022). However, there are still poorly investigated areas, as observed with the recently identified species within the *Potamophylaxwinneguthi* species group in the Western Balkans. In the span of a decade, the recognised species within this group have surged from three to eleven, a revelation derived from both morphological and molecular analyses (Ibrahimi et al. 2021b, Ibrahimi et al. 2022, Ibrahimi et al. 2023).

This distinctive species group is exclusively localised to Albania, Bosnia and Herzegovina, Bulgaria, Kosovo, North Macedonia and Serbia (Kumanski and Malicky 1999, Oláh and Kovács 2012, Oláh and Kovacs 2014, Ibrahimi and Bilalli 2021, Ibrahimi et al. 2021b, Ibrahimi et al. 2022, Ibrahimi et al. 2023). All members of this group exhibit shared ecological features and distribution patterns, predominantly being found in spring areas and upstream segments of mountainous streams and rivers. Their distributional range exhibits an exceptionally narrow area, at times restricted to a solitary stream or river. Furthermore, adults of these species display activity for only a few weeks during the autumn season. Two species clusters are known within this species group, the *tagas* cluster and the *winneguthi* cluster.

An intriguing aspect of these caddisflies is their reported sensitivity to habitat degradation, pollution and alterations in water regime and characteristics, particularly attributed to the presence of hydropower plants (Ibrahimi et al. 2016, Ibrahimi et al. 2024) and other human-induced deteriorations.

A few years ago, the molecular analysis of specimens from a population belonging to the *Potamophylaxwinneguthi* species cluster in the northern part of the Republic of Kosovo revealed significant differences from the other species in this cluster (Ibrahimi et al. 2021). However, due to the limited number of male specimens, morphological analysis was not concluded at that time. In 2022 and 2023, during a study on reference sites and conditions in the Ibër Basin in the Republic of Kosovo, extensive sampling was conducted, resulting in a substantial number of male specimens. Following a comprehensive morphological analysis, we now identify it as a new species, named *Potamophylaxkosovaensis* sp. nov.

## Materials and methods

*Fieldwork and laboratory analysis.* Adults of the new species were collected using ultraviolet light traps, adhering to the methodology outlined by Malicky (2004) for nocturnal light trapping. Subsequently, these specimens were preserved in 90% ethanol and are presently deposited at the Department of Biology, Faculty of Mathematics and Natural Sciences, University of Prishtina “Hasan Prishtina” in Prishtinë, Kosovo.

To assess the morphological features of *Potamophylaxkosovaensis* sp. nov., we used specimens of *Potamophylaxjuliani* Kumanski, 1999, *Potamophylaxwinneguthi* Klapalek, 1902, *Potamophylaxidliri* Ibrahimi, Bilalli & Kučinić, 2022, *Potamophylaxcoronavirus* Ibrahimi, Bilalli & Vitecek, 2021 and *Potamophylaxhumoinsapiens* Ibrahimi & Bilalli, 2023. These specimens were obtained from Osogovo Mountain in Bulgaria, Zlatibor Mountain in Serbia, Jastrebac Mountains in Serbia, Bjeshkët e Nemuna in the Republic of Kosovo and Sharr Mountains in the Republic of Kosovo, respectively. For species that were unavailable (*Potamophylaxhaidukorum* Malicky, 1999), comparative assessments was conducted, based on existing literature (Kumanski and Malicky 1999, Malicky 2004).

Morphological analysis of the male terminalia of the new species involved specimens treated with 10% potassium hydroxide (KOH). The nomenclature for male terminalia adheres to Nielsen (1957) for *Limnephilusflavicornis* (Fabricius, 1787) and Kumanski and Malicky (1999). Additionally, systematic nomenclature follows Morse (2023).

For the analysis of the genitalia features of *Potamophylaxkosovaensis* sp. nov., we utilised 15 male specimens, employing an Olympus SZX16 stereomicroscope. The resulting illustrations were prepared in Adobe Illustrator (version Creative Cloud 2018) by digitising pencil templates derived from pictures taken with the Olympus SC50 camera.

*Sampling area.* The Ibër River, spanning eastern Montenegro, northern Kosovo and central Serbia, boasts a total length of 272 km. Originating in the Hajla Mountain of Rozhajë in eastern Montenegro, the River winds its way through south-western Serbia and northern Kosovo before re-entering Serbia, ultimately merging with the West Morava River near Kraljevo in central Serbia. The Ibër River Basin holds the distinction of being the second-largest in Kosovo, encompassing a network of several significant rivers that contribute to its hydrological complexity. Amongst these rivers are the Llap River, Sitnica River, Bistrica River and Kaçandoll River which are the most significant ones (Ibrahimi et al. 2018, Bulliqi 2000).

The specimens of the new species were collected at two distinct locations within the Kopaonik Mountains, falling under the jurisdictions of the Podujevë and Mitrovicë Municipalities in the Republic of Kosovo.

The first sampling site is situated in a spring area of a small tributary of the Kaçandoll River in the Bajgorë area. Until recently, the site was surrounded by dense vegetation, but over the past few years, it has gradually transformed into an open stream due to deforestation in the surrounding area.

The second sampling station (Fig. 1) is located at a spring area of a tributary of the Llap River in Marincë Village. The spring itself is nestled within a beech forested area and, as it progresses, it traverses through a semi-forested region comprised of deciduous and coniferous trees. It represents one of the main contributers to the Llap River.

## Taxon treatments

### Potamophylax kosovaensis

Ibrahimi & Bilalli sp. nov.

7B5C4669-8ACA-538A-8CE8-A1C405581FAC

F3015EBD-CA77-4BBE-8A97-755F30C43A3A

#### Materials

- **Type status:** Holotype. **Occurrence:** recordedBy: Halil Ibrahimi; individualCount: 1; sex: male; lifeStage: adult; occurrenceID: 9D8B3DAF-8AE6-5057-BF54-ACABB44E7D46; **Taxon:** class: Insecta; order: Trichoptera; family: Limnephilidae; genus: Potamophylax; specificEpithet: kosovaensis; taxonRank: species; nomenclaturalCode: ICZN; **Location:** continent: Europe; waterBody: Black Sea Basin; country: Kosovo; municipality: Mitrovicë; locality: Kaçandoll; verbatimLocality: sidestream of the Kaçandoll River, on the main regional Bajgorë road; verbatimElevation: 1262; verbatimCoordinates: 42.9798N, 21.05098E; verbatimCoordinateSystem: decimal degrees; **Event:** samplingProtocol: entomological net; samplingEffort: 11 observer-hours; eventDate: 11/11/2023; year: 2013; month: 11; day: 11
- **Type status:** Paratype. **Occurrence:** recordedBy: Halil Ibrahimi; individualCount: 1; sex: male; lifeStage: adult; occurrenceID: FB028093-AD4E-55FC-BCBC-EBCD607EBA43; **Taxon:** class: Insecta; order: Trichoptera; family: Limnephilidae; genus: Potamophylax; specificEpithet: kosovaensis; taxonRank: species; nomenclaturalCode: ICZN; **Location:** continent: Europe; waterBody: Black Sea Basin; country: Kosovo; municipality: Mitrovicë; locality: Kaçandoll; verbatimLocality: sidestream of the Kaçandoll River, on the main regional Bajgorë road; verbatimElevation: 1262; verbatimCoordinates: 42.9798N, 21.05098E; **Event:** samplingProtocol: entomological net; samplingEffort: 11 observer-hours; eventDate: 11/11/2023; year: 2013; month: 11; day: 11
- **Type status:** Paratype. **Occurrence:** recordedBy: Halil Ibrahimi, Astrit Bilalli; individualCount: 25; sex: male; lifeStage: adult; occurrenceID: 8AD4E237-0ED8-5308-B316-EEF80F275D0B; **Taxon:** class: Insecta; order: Trichoptera; family: Limnephilidae; genus: Potamophylax; specificEpithet: kosovaensis; taxonRank: species; nomenclaturalCode: ICZN; **Location:** continent: Europe; waterBody: Black Sea Basin; country: Kosovo; municipality: Podujevë; locality: Marincë; verbatimLocality: Kroi i Konakut spring; verbatimElevation: 1410; verbatimCoordinates: 43.111556N, 21.003333E; **Event:** samplingProtocol: UV light trap; samplingEffort: 3 trap nights; eventDate: 14/11/2023; year: 2023; month: 11; day: 14

#### Description

**Figure 2.**
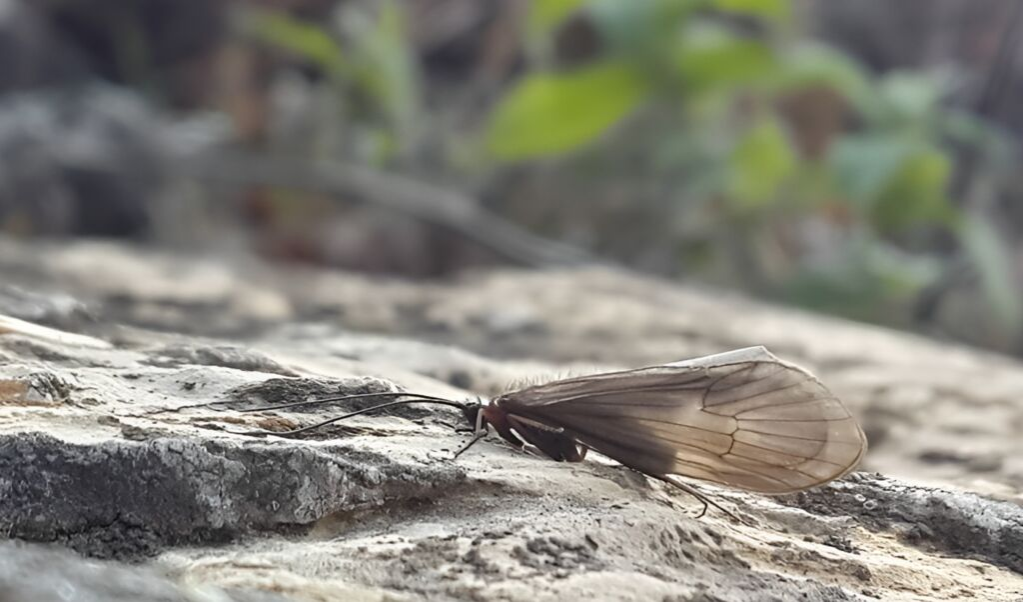
Male of *Potamophylaxkosovaensis* sp. nov. photographed at the type locality.

Male. General appearance (Fig. 2). Habitus generally brown, prothorax, sclerites of meso- and metathorax, coxae and femora dark brown to brown; tibiae and tarsi brown. Wings brown with brown setae. Male maxillary palps 3-segmented. Forewing length 14.5–15.8 mm. Spur formula 1-3-4. Antennae brown.

Male genitalia (Figs 3, 4, 5). Tergite VIII generally brown with darker patches, in dorsal view roughly rectangular, with apical portion slightly narrower; several irregularly distributed setae of different sizes concentrated on proximal sclerotised portion of segment VIII; spinate area located on semi-membranous distal portion of segment VIII with a slightly wider proximal portion in dorsal view, elongated, covered by small black spines. Segment IX light brown with few darker patches mostly on the edges, laterally broad, anterior margin convex, with narrow dorsal and ventral portions. Superior appendages light brown; in lateral view, long, subrectangular, base narrower than the apex, bearing rounded tips and covered with medium-sized, thin setae. Intermediate appendages, sickle-shaped with accuminate apex, turned upwards. Inferior appendages, short, symmetric, with bifid apex, directed mesad, each edges pointed, with longer ventral edge. Phallic apparatus consists of small aedeagus and a pair of parameres; aedeagus bulbous in ventral view, mainly wide at tip and basally, bifid rounded apex, apicomesal excision narrow-U-shaped; parameres long and moderately wide, equally wide along the entire length in lateral and ventral views, bearing 10-17 long, thick spines distributed apicodorsally.

#### Diagnosis

Males of the new species can be easily distinguished from those of other species in the *Potamophylaxwinneguthi* species group by their small aedeagus and the presence of thick apical spines on the parameres. The aedeagus, being the smallest in the entire species group, also differs in shape from its closest relatives, *P.humoinsapiens* and *P.idliri*, when viewed laterally and ventrally. The thick apical spines on the parameres of the new species are unique within the group, except for *P.winneguthi*, although, in the latter species, the spines are much thicker and longer, with other notable differences in the shape of the inferior appendages and aedeagus. The spines in the new species are evenly distributed apicodorsally in a regular manner, distinctly separated from each other. In contrast, in *P.humoinsapiens*, the spines are thin and hair-like, while, in *P.idliri*, they are thinner than in *P.kosovaensis* sp. nov. and distributed only apically, with the medium ones being the longest. Additionally, the parameres of the new species maintain a consistent thickness along their entire length, whereas, in *P.idliri*, they are generally thinner, thickest apically and, in *P.humoinsapiens*, they are thick along the basal half and thin along the remaining length.

#### Etymology

The species name is derived from the Latinised adjectival form "kosovaensis," signifying its association with the Republic of Kosovo (in Albanian language “Kosova”), the state where it was found.

#### Distribution

The species is most probably a microscale endemic of the Kopaonik Mountain.

#### Ecology

The new species is found exclusively at the spring areas of both streams, indicating a strict eucrenal preference. The substrate of the stream close to the sampling site at the Kroi i Konakut spring was dominated by meso- to macrolithal substrate, surrounded by dense riparian vegetation. The same substrate was observed at the second site in the Kaçandoll stream, but the riparian vegetation was considerably scarce due to illegal logging. The species was captured only by ultraviolet light traps. The species was collected during October and November, implying it has an autumn flying period.

## Discussion

Kopaonik is a mountain range stretching along the border line between the Republic of Kosovo and Serbia. Currently, only few data are available regarding the diversity of caddisflies in this mountain range, with most of them coming from the Kosovan part. *Potamophylaxkosovaensis* sp. nov. represents the second known endemic caddisfly species exclusive to the Kopaonik Mountains, with the first being *Drususdardanicus* Ibrahimi, Kucinic & Vitecek, 2015, both of them known currently only from the Republic of Kosovo. Both species were found together in sampling station 2. It is worth noting that the distribution of species within the *Potamophylaxwinneguthi* species group often aligns with the distribution of endemic species belonging to the genus *Drusus* in the Balkan Peninsula. A similar case was noted with *P.humoinsapiens* and *Drusussharrensis* Ibrahimi, Vitecek & Previšić, 2016 in the Sharr Mountains, then *P.coronavirus* with *D.krusniki* Malicky, 1981 in Bjeshkët e Nemuna, as well as *P.juliani* with *D.osogovicus* Kumanski, 1982 in Osogovo Mountain (Kumanski 1988, Kumanski and Malicky 1999, Ibrahimi et al. 2015, Ibrahimi et al. 2016, Ibrahimi et al. 2021b). Both groups inhabit isolated habitats in mountainous spring and upstream areas at high altitudes. However, in contrast to the above-mentioned endemic species of the genus *Drusus*, which are primarily confined to spring areas, some species of the *Potamophylaxwinneguthi* species cluster, for example, *P.idliri* and *P.humoinsapiens*, are also found in lower stream segments, occasionally overlapping with endemic species of the genus *Drusus* (Previšić et al. 2014, Ibrahimi et al. 2014, Gashi et al. 2015, Ibrahimi et al. 2016, Hinić et al. 2020). On the other hand, although we sampled lower sections of streams, we only found *P.kosovaensis* sp. nov. within a few metres around the spring area.

Morphological analysis of male genitalia, based on a high number of specimens, confirms the stability of distinguishing characters of *Potamophylaxkosovaensis* sp. nov. Additionally, genetic differentiation, determined through analysis of the cytochrome c oxidase subunit I gene (COI) barcode region sequencing, aligns with the observed morphological distinctions amongst species within the *Potamophylaxwinneguthi* species group. The molecular analyses conducted by Ibrahimi et al. (2021b) and Ibrahimi et al. 2022 indicate that the most closely-related species to *P.kosovaensis* sp. nov., referred to as *Potamophylax* sp. Bajgorë in their analysis, is a population from the Rila Mountains in Bulgaria, with a p-distance of 4.7%. However, the status of this Rila species remains unresolved due to limited sampling. Initial analysis shows notable differences between *P.kosovaensis* sp. nov. and the Rila species, particularly in shorter and thicker parameres and shorter and thinner apical spines in the latter. Furthermore, the p-distance between *P.kosovaensis* sp. nov. and two other morphologically closely-related species within this cluster is significantly higher, measuring 5.3% with *P.idliri* and 6.6% with *P.humoinsapiens*.

Microendemic freshwater aquatic insect species, often confined to small and isolated habitats within specific geographic regions, face heightened vulnerability in the face of environmental changes in the Balkan Peninsula. These specialised insects, adapted to distinct ecological niches, are particularly sensitive to alterations in water quality, habitat degradation and shifts in hydrological patterns. Their limited distribution makes them more susceptible to localised threats, including pollution, habitat destruction and climate change impacts. The interplay of these factors can lead to a rapid decline in population size and, in some cases, the outright extinction of these microendemic species. Conservation efforts for such species must prioritise the preservation and restoration of their unique habitats, coupled with proactive measures to mitigate the broader environmental pressures that contribute to their vulnerability. In assessing *Potamophylaxkosovaensis* sp. nov., we noted the severe degradation of forested areas around its designated type locality, which now largely lies in ruin, with the stream itself serving as a dumping ground for garbage. Despite the challenges posed by this deteriorated environment, we deliberately chose this locality as type locality, even though the recent sampling efforts in the past years have failed to yield any specimens. The only recorded specimens from this area date back to 2013, involving the collection of just two males. This intentional decision aims to underscore the urgent need for heightened awareness regarding the destructive impacts of human activities on these rare habitats. Conversely, a nearby second locality harbouring the population of *Potamophylaxkosovaensis* sp. nov. appears to remain stable and unaffected by anthropogenic pressures, likely attributed to the site's inaccessibility in the absence of proper roads. Addressing the challenges faced by these microendemic species involves not only understanding their ecological requirements, but also advocating for the preservation of their habitats in the face of human-induced threats.

The new species exhibits characteristics of a habitat specialist, predominantly confined to specific environments, such as spring areas and the upstream segments of pristine streams. This specialisation renders it a distinctive and indicative species, particularly within the context of the Ibër Basin. The exclusive association with these particular habitats positions the species as a valuable indicator, offering insights into the ecological health and conditions of such environments in the region. The composition of aquatic bioindicator fauna at such spring habitats is still largely unknown in Kosovo, making impossible usage of these areas as reference sites in the water quality assessment studies. We found *Potamophylaxkosovaensis* sp. nov. in sympatry with dozens of species. While most of these species were not found to be strict eucrenal species (*Potamophylaxpallidus* Klapalek, 1899, *Rhyacophilatristis* Pictet, 1834, *Micropternacaesareica* Schmid, 1959, *Wormaldiasubterranea* Radovanovic, 1932 and *Wormaldia* sp.), a few of them were only found at eucrenal sites, such as: *Chaetopteryxbosniaca* Marinkovic-Gospodnetic, 1959, *Chaetopteryx* sp. and *Drususdardanicus* Ibrahimi, Kučinić & Vitecek, 2015. We have also collected larvae simultanously in order to contribute to the water quality assessment purposes. However, considering that there are many species with undescribed larval stages in the Balkans, linking larvae with adults will require additional studies, including molecular ones. In this context, this study contributes to the knowledge of possible reference sites for Ibër River Basin in Kosovo. Such findings emphasise the vulnerability of this species' group to environmental changes, underscoring the importance of rigorous conservation efforts to preserve these ecologically unique and sensitive caddisflies in the Western Balkans. In this regared, the increase of knowledge on aquatic insect taxonomy in Kosovo and Western Balkans will improve the usage of these bioindicator animals for freshwater quality assessment purposes.

## Supplementary Material

XML Treatment for Potamophylax kosovaensis

## Figures and Tables

**Figure 1a.**
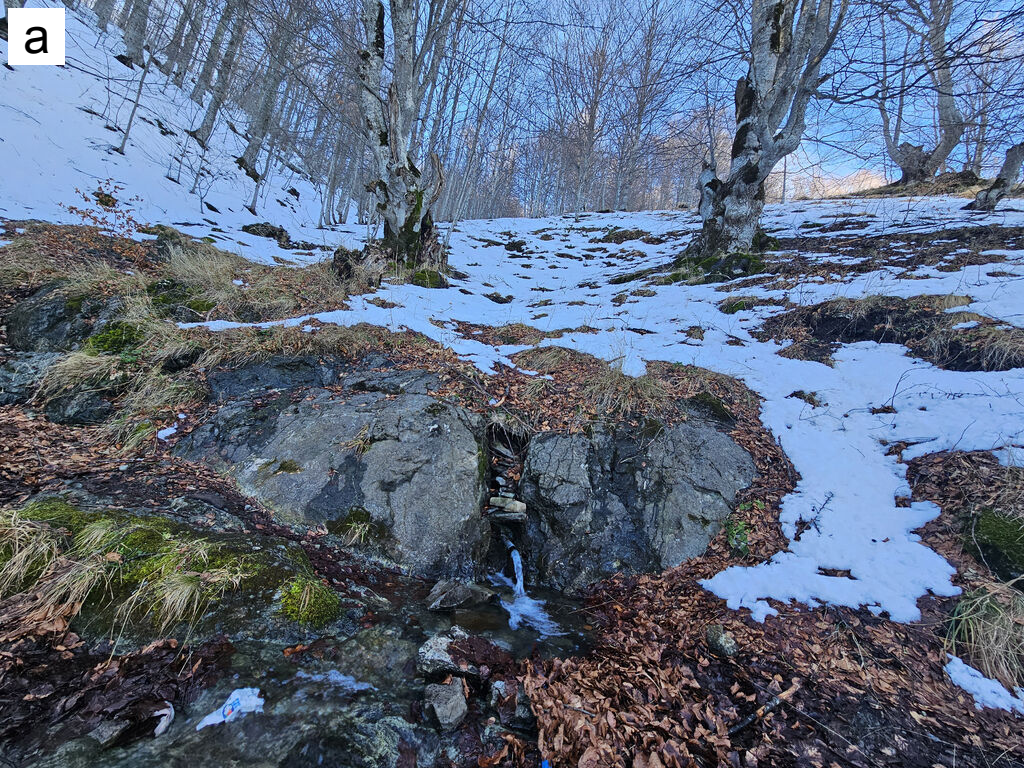
the spring;

**Figure 1b.**
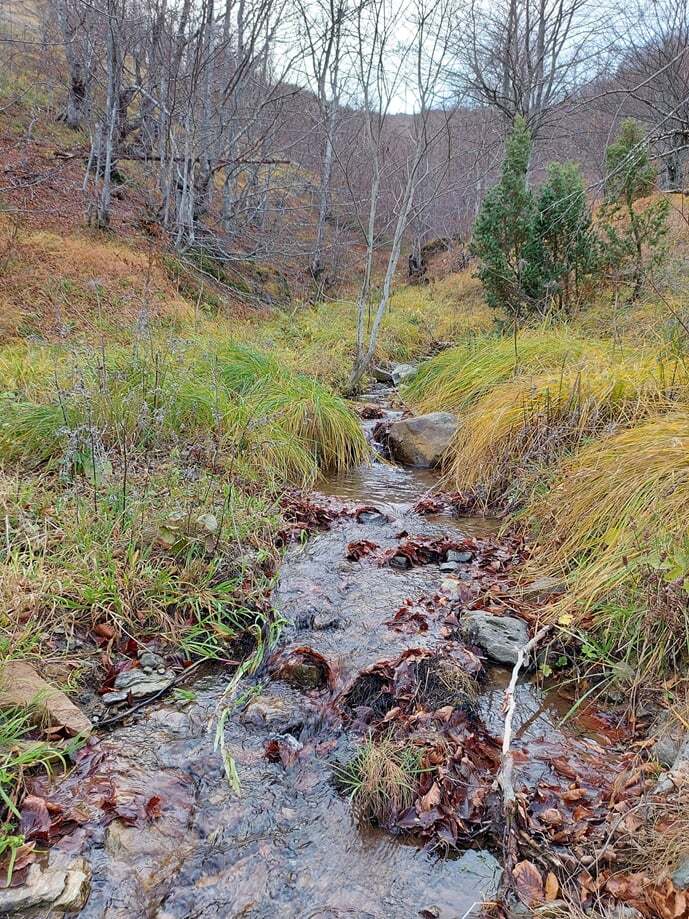
the stream.

**Figure 3a.**
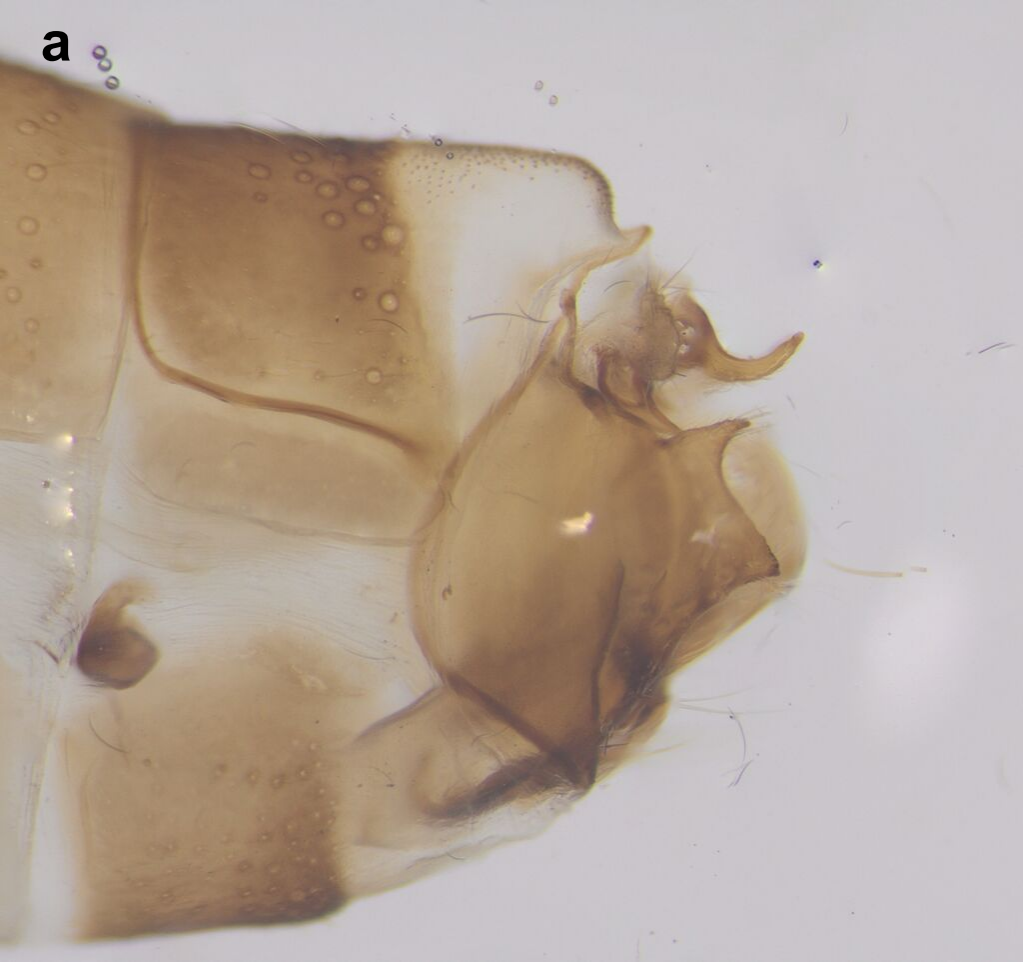
Left lateral view;

**Figure 3b.**
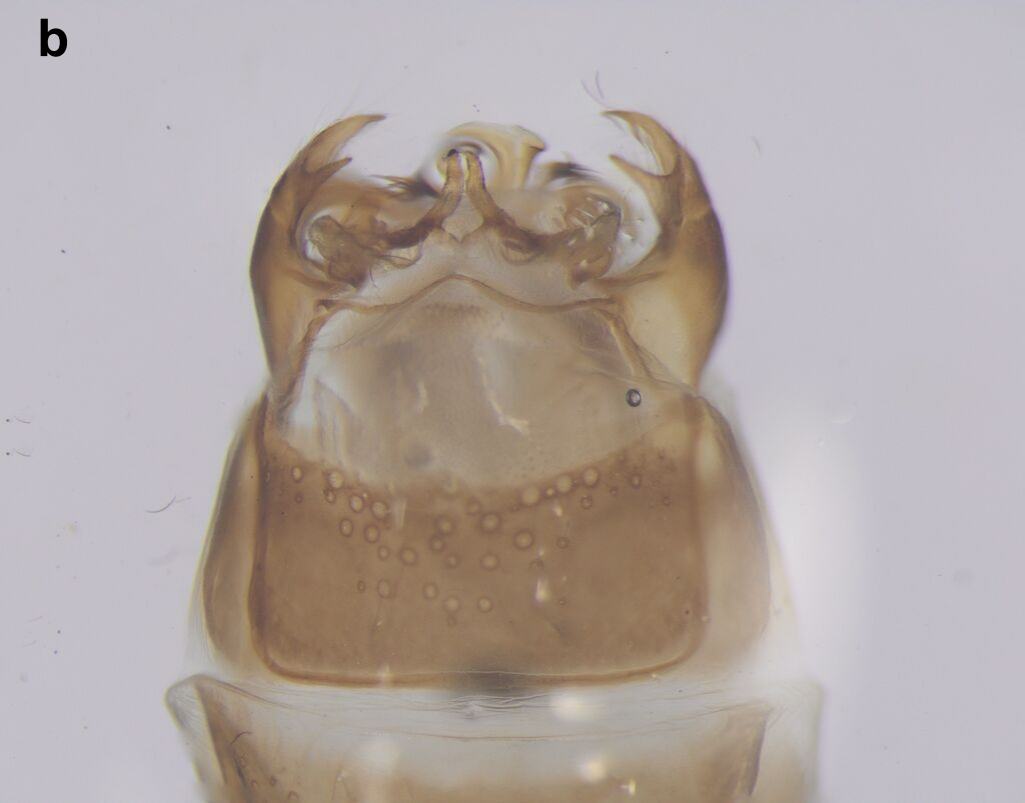
Dorsal view.

**Figure 4a.**
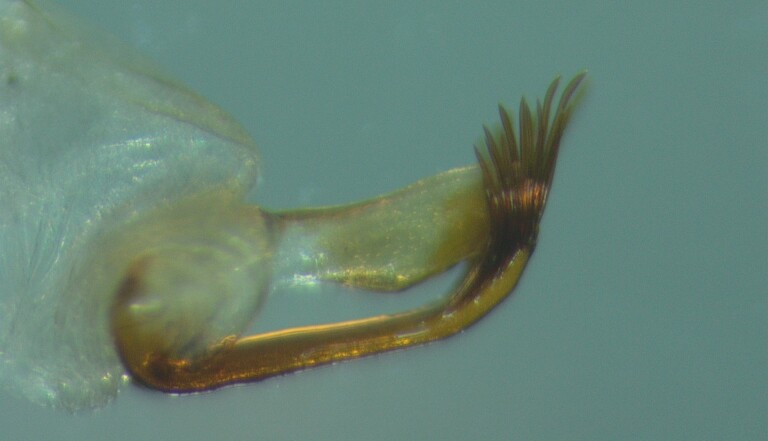
Left lateral view;

**Figure 4b.**
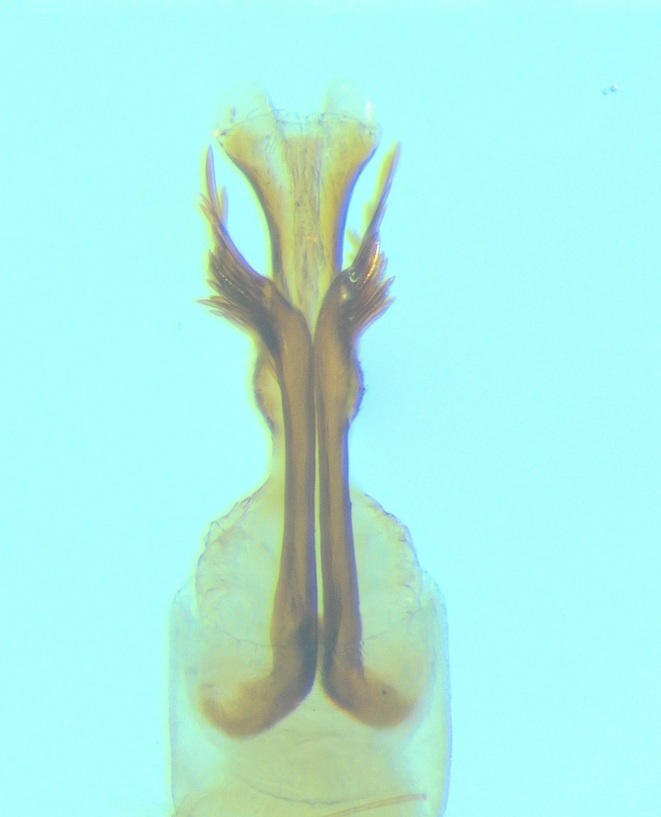
Ventral view;

**Figure 4c.**
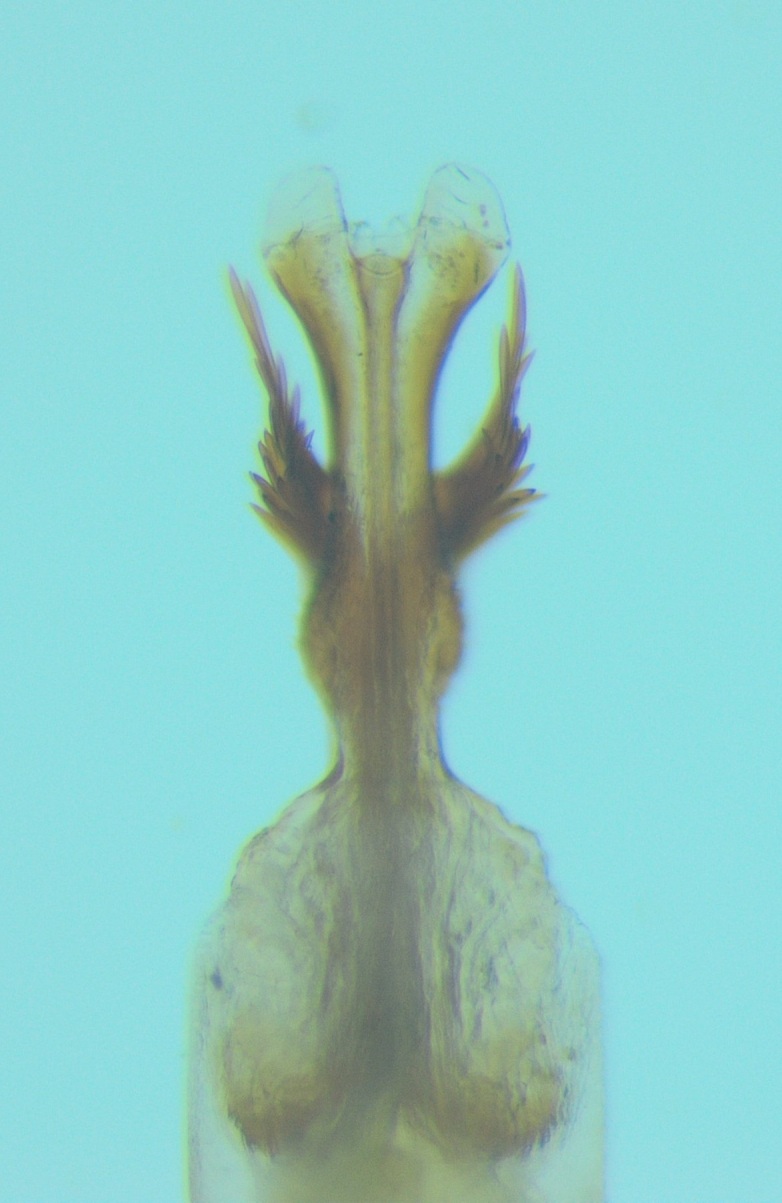
Dorsal view.

**Figure 5a.**
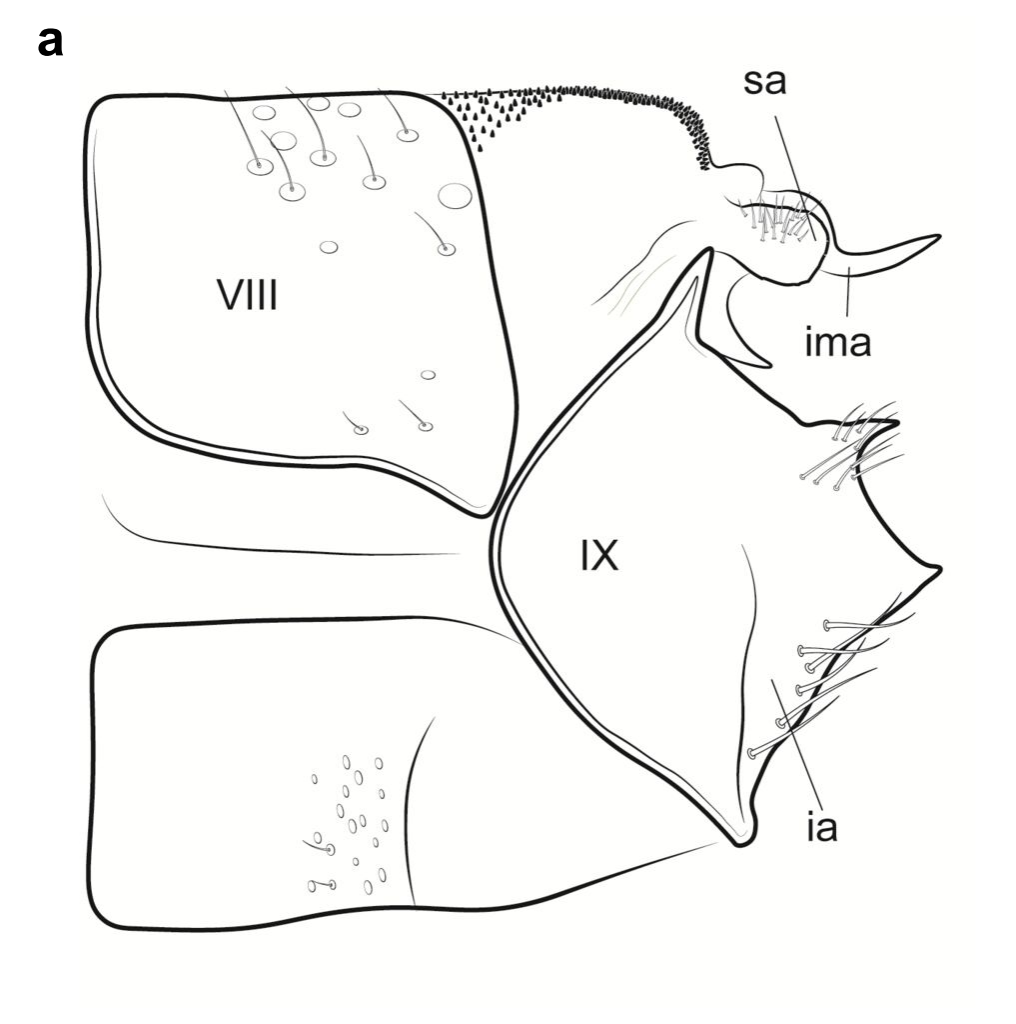
Left lateral view;

**Figure 5b.**
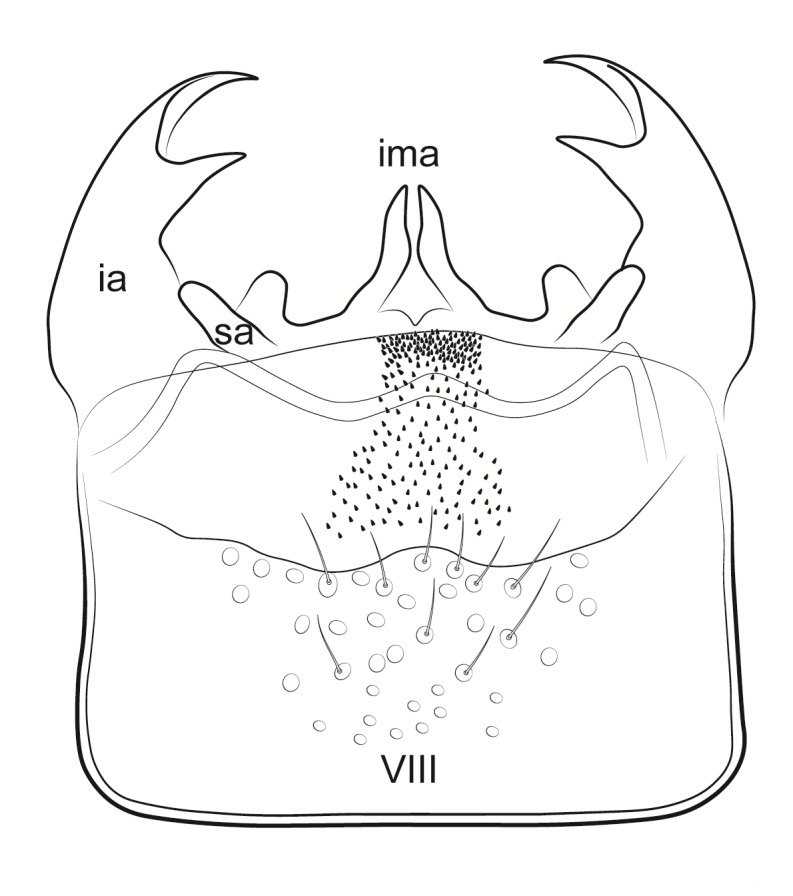
Dorsal view;

**Figure 5c.**
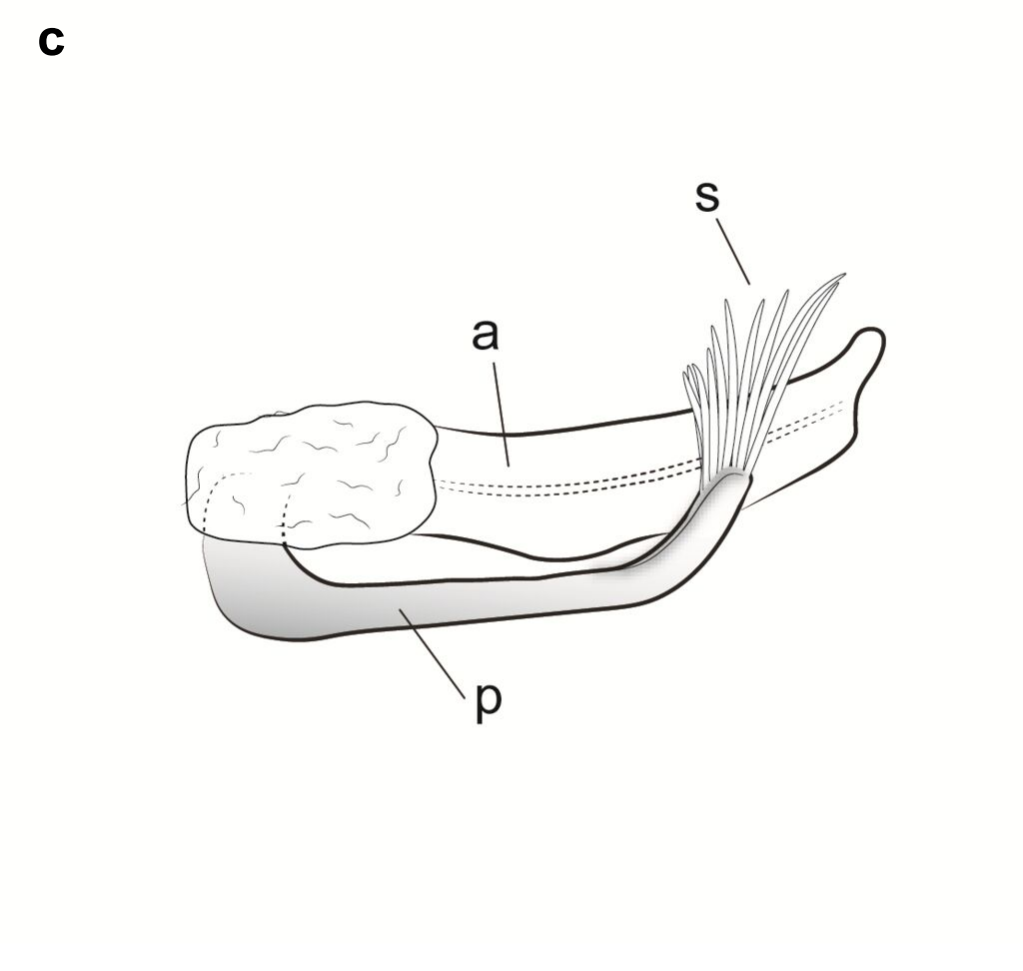
Aedeagus and parameres, left lateral view;

**Figure 5d.**
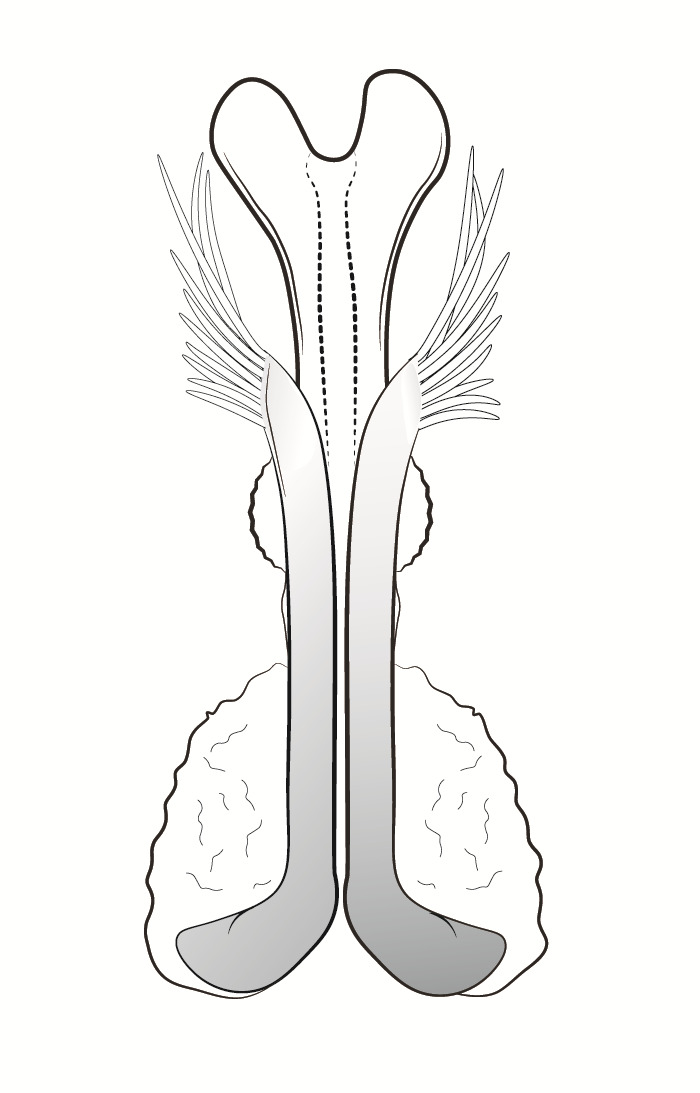
Aedeagus and parameres, ventral view.
